# Electrospun MoS_2_-CNTs-PVA/PVA Hybrid Separator for High-Performance Li/FeS_2_ Batteries

**DOI:** 10.3390/polym16070921

**Published:** 2024-03-27

**Authors:** Sheng Wu, Qian Liu, Wei Zhang, Ruizhe Wu, Hongping Tang, Yulin Ma, Wenqiang Xu, Shufang Jiang

**Affiliations:** Collaborative Innovation Center for Advanced Organic Chemical Materials Co-Constructed by the Province and Ministry, Ministry of Education Key Laboratory for the Synthesis and Application of Organic Functional Molecules, College of Chemistry and Chemical Engineering, Hubei University, Wuhan 430062, China; 202321106011521@stu.hubu.edu.cn (S.W.); 202221106011729@stu.hubu.edu.cn (Q.L.); 202131106030105@stu.hubu.edu.cn (W.Z.); 202131106030099@stu.hubu.edu.cn (H.T.); 202231106030023@stu.hubu.edu.cn (Y.M.); 202221113012384@stu.hubu.edu.cn (W.X.)

**Keywords:** electrospinning, polyvinyl alcohol, MoS_2_, Li/FeS_2_ batteries

## Abstract

As a promising candidate for high-energy-density rechargeable lithium metal batteries, Li/FeS_2_ batteries still suffer from the large volume change and severe shuttle effect of lithium polysulfides during cycling. To improve the electrochemical performance, great efforts have been made to modify FeS_2_ cathodes by constructing various nanocomposites. However, energy density is sacrificed, and these materials are not applicable at a large scale. Herein, we report that the electrochemical performance of commercial FeS_2_ can be greatly enhanced with the application of a double-layer MoS_2_-CNTs-PVA (MCP)/PVA separator fabricated by electrospinning. The assembled Li/FeS_2_ batteries can still deliver a high discharge capacity of 400 mAh/g after 200 cycles at a current density of 0.5 C. The improved cycling stability can be attributed to the strong affinity towards lithium polysulfides (LiPSs) of the hydroxyl-rich PVA matrix and the unique double-layer structure, in which the bottom layer acts as an electrical insulation layer and the top layer coupled with MoS_2_/CNTs provides catalytic sites for LiPS conversion.

## 1. Introduction

With the fast development of lithium-ion battery (LIB) technologies in recent years, the energy density and cycling stability of LIBs have been significantly improved. However, the LIBs based on conventional cathode materials, including LiCoO_2_, LiFePO_4_ and nickel-rich-layered oxides (LiNi_x_Mn_y_Co_z_O_2_, x ≥ 0.7), and Si/C composite anodes have difficulty achieving a cell-level energy density beyond 500 Wh/kg [1]. Unlike the above intercalation-type cathode materials, conversion-type cathodes (MX_n_; M = Fe, Co, Cu, none, etc.; X = S, F, Cl, O, etc.) can store more lithium ions by forming Li_m_X_n_ compounds, which can provide a significantly higher specific capacity (for example, 1673 mAh/g for S) than the current state-of-the-art lithium-rich manganese oxides (300 mAh/g) and greatly enhance the energy density when paired with lithium metal anodes [2,3]. Among those conversion-type cathode materials, pyrite FeS_2_ is attractive due to its high specific capacity (894 mAh/g) and good electrical conductivity (10^−2^ S/cm compared with sulfur’s 10^−30^ S/cm), which enables Li/FeS_2_ batteries to simultaneously achieve a high energy density and a high power density [2,4]. Furthermore, since pyrite FeS_2_ is abundant in the earth and has a low cost, it has been widely employed in primary batteries. However, like other conversion-type cathode materials, FeS_2_ suffers from the huge volume change during charge/discharge cycles which leads to the collapse and detachment of FeS_2_ particles from the conductive matrix [5,6]. Additionally, since sulfur is formed and participates in the electrochemical process after the first cycle, the shuttle effect of the produced lithium polysulfides (LiPSs) aggravates the cycling performance of Li/FeS_2_ batteries [7,8,9,10].

To handle these problems, an effective approach is to synthesize nanostructured FeS_2_ particles with carbon shells, which can enhance electrical conductivity, buffer the volume expansion and prevent the dissolution of polysulfides [11,12]. However, such modifications will inevitably reduce the FeS_2_ content in cathodes, thus sacrificing the overall energy densities of the batteries, which is not feasible for practical applications. What is even worse is that significant irreversible capacity loss in the first few cycles usually occurs even with compact carbon shells [13,14]. The modification of the cathode–electrolyte interphase (CEI) with electrolyte optimization or ionic liquid is another effective strategy; however, it is still difficult to apply to commercial FeS_2_ materials directly [15,16]. Recently, Wang and coworkers constructed a stable ionic–electronic network in FeS_2_ cathodes by electrolyte and binder optimization and achieved a capacity retention of more than 70% after 700 cycles at a current rate of 0.5 C with commercial FeS_2_ materials for the first time [17]. One of the key points of their findings is the inhibition of the shuttle effect with a low-LiPS-solubility electrolyte. Therefore, it is also possible to achieve enhanced cycling performance of Li/FeS_2_ batteries by mitigating the shuttle effect with separator optimization. In this regard, modifications of commercial polyolefin separators have been widely applied to alleviate the shuttle effect of LiPSs for sulfur and transition metal sulfide cathodes [18,19]. Various materials, such as polyethylene oxide (PEO) [20,21,22], g-C_3_N_4_ [23], MoS_2_ [24] and metal–organic frameworks (MOFs) [25], have been applied on separators to adsorb lithium polysulfides via vacuum filtration and the solution casting method. Nonetheless, performance is still restricted by the pristine nanostructure of commercial separators, and the coating materials may block the channels for lithium-ion diffusion without careful design [26]. Separators fabricated by electrospinning are attractive due to their ideal features like interconnected porous structures and large surface-to-volume ratios [27]. Among various polymers, the application of polyvinyl alcohol (PVA) in separators has been demonstrated and the hydroxyl-rich surface of PVA is believed to absorb LiPSs strongly [28,29,30], which is beneficial for the cycling stability of Li/FeS_2_ batteries.

In this work, we fabricated double-layer PVA-based hybrid films (MoS_2_-CNTs-PVA/PVA) by the electrospinning technique and aimed to apply them as separators in Li/FeS_2_ batteries based on commercial FeS_2_ materials. The highly porous structure was characterized by SEM and facilitated fast lithium-ion transport with excellent electrolyte wettability. The cycling performances of the Li/FeS_2_ batteries assembled with the hybrid separator exhibited a high capacity of 400 mAh/g after 200 cycles at a current density of 0.5 C. The improved cycling stability can be attributed to the strong affinity towards LiPSs of the hydroxyl-rich PVA matrix and the unique double-layer structure, in which the bottom layer acts as an electrical insulation layer and the top layer combined with MoS_2_/CNTs provides catalytic sites for LiPS conversion. Our findings provide a simple and effective way to improve the cycling performance of Li/FeS_2_ batteries based on commercial FeS_2_ materials.

## 2. Experiments

### 2.1. Materials

FeS_2_ was purchased from Yunfu Huana New Materials Technology Co., Ltd., Yunfu, China. Poly(vinyl alcohol) (PVA, 1788, degree of hydrolysis: 87.0~89.0 mol%) was obtained from Shanghai Macklin Biochemical Technology Co., Ltd., Shanghai, China. MoS_2_ powders and N-methyl-2-pyrrolidone (NMP) were obtained from Shanghai Aladdin Biochemical Technology Co., Ltd., Shanghai, China. Carbon nanotubes were obtained from Nanjing XFNANO Materials Tech Co., Ltd., Nanjing, China. PVDF (HSV900), a commercial PP separator (Celgard 2500) and Super P were obtained from Canrd, Dongguan, China. The electrolyte used for electrochemical measurements was 1M LiTFSI in 1:1 *v*/*v* DOL/DME and was obtained from Suzhou Duoduo Chemical Technology Co., Ltd., Suzhou, China. All chemicals were used without further purification or modification.

### 2.2. Preparation of MoS_2_-CNTs-PVA (MCP)/PVA Separator

The polymer nanofiber membranes were prepared by a typical two-step electrospinning process. A 10 wt% PVA solution was prepared by stirring PVA particles in deionized water at 80 °C for 16 h, which was used to electrospin the pure PVA layer and make MoS_2_-CNT-PVA slurry. In order to prepare the MoS_2_-CNT-PVA layer, MoS_2_ and CNTs were added to the 10 wt% PVA solution at a mass ratio of 1:1:20 (MoS_2_:CNTs:PVA) to produce a spinning slurry. The electrospinning was carried out in a 5 mL plastic syringe with a 20-gauge needle, and the flow rate of suspension was controlled at a feeding rate of 0.5 mL/h. The voltage of the needle was set to 9 kV by a voltage generator, and the metal plate collector was set to −3 kV by another one. The distance between the needle tip and the metal plate collector was 20 cm. After electrospinning the pure PVA layer for 2 h, the syringe was replaced by another one containing the MoS_2_-CNT-PVA slurry to be electrospun for another 2 h to obtain the final membrane. The whole electrospinning process was performed at room temperature and 45% relative humidity.

### 2.3. Characterizations

X-ray diffraction (XRD) tests were carried out by a Bruker D8 ADVANCE instrument with CuKα radiation at a scanning range of 10–80°. Raman measurements were conducted by a Renishaw InVia Raman Microscope (Renishaw, New Mills, UK) using a 532 nm excitation laser. Scanning electron microscopy (SEM) images and elemental analyses were obtained by a ZEISS Sigma 500 FESEM (Zeiss, Jena, Germany) equipped with a Bruker QUANTAX EDS (Bruker, Billerica, MA, USA). Contact angles were measured by the sessile drop method on a contact-angle meter. Electrolyte uptake was determined by the percentage change in weight after soaking the membrane in electrolytes for 1 h. For the measurement of the porosity, the separators (d = 17 mm) were first weighed in the dry state and weighed again after soaking in liquid kerosene for 12 h. The volume of adsorbed kerosene was calculated by the weight increase and the density of kerosene (0.82 g/cm^3^). The porosity of the separator was determined by the ratio of the adsorbed kerosene volume to the volume of the separator.

### 2.4. Electrochemical Measurements

Electrochemical performance was evaluated with CR2025-type coin cells assembled inside an argon-filled glove box with a water and oxygen content less than 0.1 ppm at 25 °C. An ether-based electrolyte (1 M LiTFSI in 1:1 vol/vol DOL/DME) was adopted, and 50 μL of electrolyte was added to each cell. For the assembly of symmetric cells, two pieces of stainless steel (SS) plates (d = 15.8 mm) or lithium chips (d = 14.5 mm) were separated by a piece of commercial PP separator or MCP/PVA hybrid separator with a diameter of 17 mm. Electrochemical impedance spectroscopy (EIS) measurements were taken at a voltage amplitude of 5 mV and in a frequency range of 0.001–500 kHz with a CHI 760 E. The Li/FeS_2_ cells were assembled with FeS_2_ cathodes (d = 10 mm), separators (d = 17 mm) and lithium chips (d = 14.5 mm). Commercial PP and MCP/PVA separators were adopted for comparisons. For the preparation of FeS_2_ cathodes, FeS_2_ slurry was obtained by mixing FeS_2_, PVDF and Super P in a weight ratio of 8:1:1 with NMP as the solvent. The slurry was cast onto Al foil by doctor blading and dried in an oven at 60 °C for 2 h. Then, the FeS_2_ cathodes were cut into disks with a diameter of 10 mm with an FeS_2_ loading of ~1.0 mg cm ^−2^ and further dried in a vacuum at 120 °C for 12 h before use. Charging and discharging tests were carried out on a battery test system (CT-4008, Neware, Shenzhen, China) in the voltage range of 1–3 V. The current was determined by the mass of FeS_2_ and its theoretical capacity of 894 mAh/g.

## 3. Results and Discussion

### 3.1. Characterization of the Hybrid Separator

**Figure 1:**
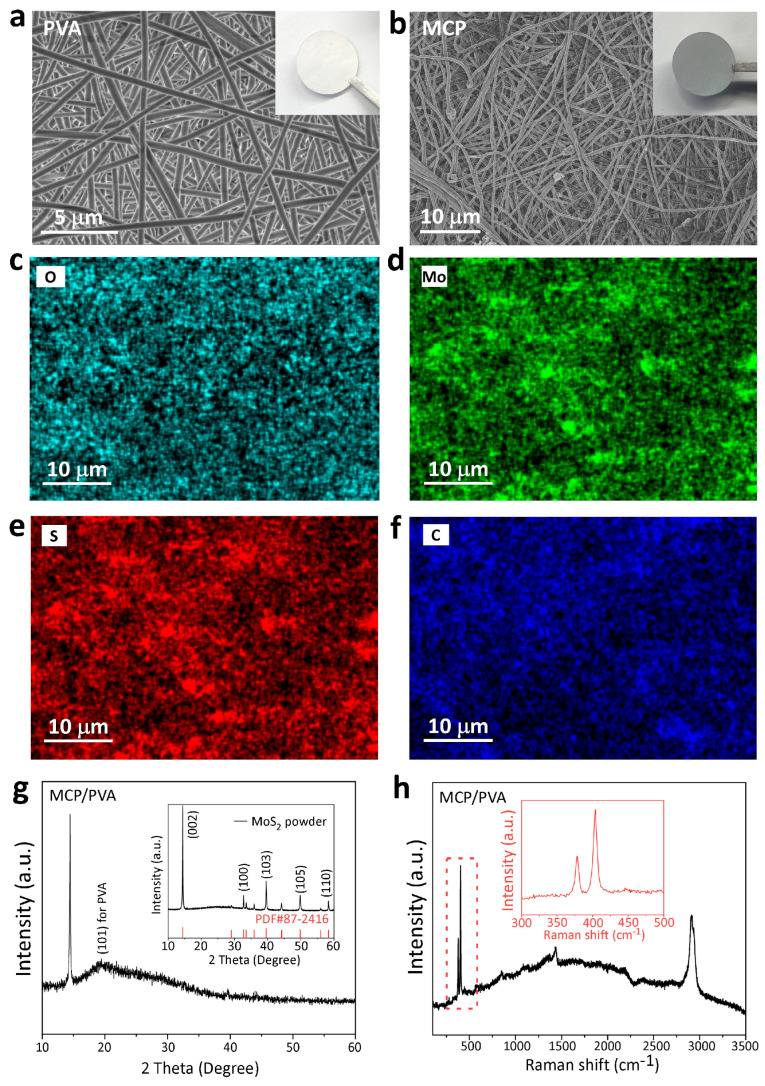
Characterizations of the fabricated MCP/PVA hybrid film. SEM images of the (**a**) PVA side and (**b**) the MCP side. (**c**–**f**) The corresponding EDS mapping results. (**g**) XRD patterns of the MCP film and MoS_2_ powders. (**h**) Raman spectra of the MCP film; the inset is the enlarged area.

The morphology and crystalline structure of the MoS_2_-CNTs-PVA (MCP)/PVA hybrid separator were characterized by scanning electron microscopy (SEM), XRD and Raman spectroscopy. As shown in the SEM image in Figure 1a, the bottom PVA layer is composed of smooth fibers with an average diameter of ca. 422 nm. When the MoS_2_ and CNTs were added into PVA to form the composite, the fibers became thinner, with an average diameter of 316 nm, as shown in Figure 1b. The reduction in the fiber diameter is due to the enhanced conductivity of the PVA solution with the addition of CNTs. In the strong electric field during electrospinning, the volume charge density of the PVA solution was increased by the enhanced conductivity, resulting in the stronger electrostatic repulsion near the nozzle, which easily overcame the surface tension and decreased the fiber diameter [31]. Considering the densely electrospun and thick PVA layer, as shown in Figure 1a, inter-diffusion of the conductive MCP layer into the PVA layer can be avoided and therefore remove the risk of a short circuit. To investigate the distribution of MoS_2_ and CNTs in the fibers, energy-dispersive X-ray spectroscopy (EDS) analysis was conducted. Based on the elemental mapping results in Figure 1c–f, the signals of all the main elements are relatively uniform in the whole area. However, a few large aggregates can still be found in some areas (Figure S1), and the elemental analysis showed a much stronger O signal, indicating the accumulation of PVA or some other impurities, which will not raise the risk of short circuit. Therefore, it can be concluded that the MoS_2_ and CNT additives are uniformly dispersed and wrapped by the fibers, forming an interconnected three-dimensional network, which can provide abundant adsorption sites for the LiPSs and favor the transport of lithium ions. To determine the crystallinity of the MCP/PVA hybrid separator, XRD characterizations were conducted. As shown in Figure 1g, one strong peak at 2θ = 15° and one small peak at 2θ = 40° for MoS_2_ can be identified, which matches well with the spectrum of the MoS_2_ powder (PDF#87-2416), as shown in the inset. In addition, a small peak located at 2θ = 19.5° can be attributed to the (101) plane of PVA [32]. Raman spectroscopy was also used to determine the composite structures. In Figure 1h, two peaks at 1418 cm^−1^ and 2911 cm^−1^ can be identified for the PVA matrix. The existence of MoS_2_ can be confirmed by the peaks at 378 cm^−1^ and 403 cm^−1^, as shown in the magnified inset image.

### 3.2. Electrolyte Wettability

**Figure 2:**
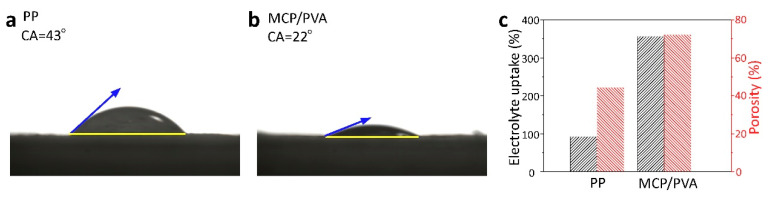
Contact-angle measurements of (**a**) PP and (**b**) MCP/PVA. (**c**) Porosity and electrolyte uptake results for both separators.

To apply the MCP/PVA hybrid film as the separator for Li/FeS_2_ batteries, a good electrolyte wettability is required. Therefore, contact-angle measurements were conducted to evaluate the electrolyte affinity for different separators. Since commercial PP, PE and PE/PP/PE separators are all polyolefin separators produced by stretching methods and have similar surface properties, PP separators were adopted for comparison with the electrospinning hybrid separators in this work. According to the contact-angle testing results in Figure 2a,b, the electrolyte on the MCP/PVA hybrid separator exhibits a much smaller contact angle of ~22° compared with that of commercial PP separators (~43°). As shown in Figure S2, the electrolyte contact angle on the pure PVA membrane was measured to be ~23°, which suggests that the improved electrolyte wettability was mainly due to the PVA network [29,33]. The high electrolyte affinity of the hybrid separator can be ascribed to the abundance of hydroxyl groups in PVA chains, which have strong interactions with the ether-based electrolytes. Moreover, the strong capillary force induced by the highly porous nanostructure of the fabricated hybrid separator is also beneficial for the electrolyte wettability and adsorption. Additionally, porosity and electrolyte uptake measurements were also carried out. As depicted in Figure 2c, it was found that the porosity of the MCP/PVA hybrid separator and commercial PP separators was 72% and 44%, respectively. The much higher porosity of the MCP/PVA hybrid separator leads to a larger saturation electrolyte uptake of 356% compared with the PP separator (92%) after soaking in electrolytes for 1 h. Due to the greatly improved electrolyte wettability, it is expected that the electrochemical performance of the hybrid separator will be enhanced.

### 3.3. Electrochemical Performance

**Figure 3:**
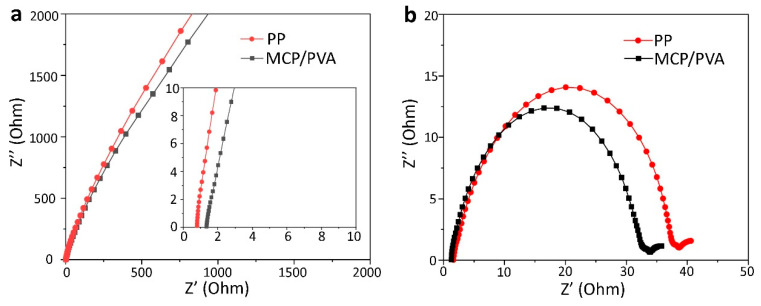
EIS measurements of (**a**) SS/separator/SS (the inset is the magnified image near the intersection points) and (**b**) Li/separator/Li symmetric cells.

The ionic conductivity of the MCP/PVA hybrid separator and the commercial PP separator was measured with SS/separator/SS symmetric cells at 30 °C. Figure 3a shows Nyquist plots of the cells, and the intercept on the Z’ axis in the high-frequency range refers to the bulk resistance (R_b_) of the separator. It can be observed that the R_b_ values of the PP and the MCP/PVA hybrid separators are 0.79 Ω and 1.34 Ω, respectively. Considering the thickness of both separators (PP: 25 μm; MCP/PVA: 60 μm) and the diameter of SS (15.8 mm), the ionic conductivities of the PP and the MCP/PVA hybrid separators were calculated to be 1.79 mS/cm and 2.53 mS/cm. The high ionic conductivities of the hybrid separator also validate that incorporation of the MoS_2_ and CNT into the PVA matrix does not block the channels for lithium-ion transfer, which is important for the application in batteries. In addition, the interfacial compatibility between the separator and the lithium metal was also investigated by the EIS study on Li/separator/Li symmetry cells, and Figure 3a shows the corresponding Nyquist plots. Similar to the SS/separator/SS situation, the R_b_ values of both cells are close, while the charge transfer resistance (R_ct_, the diameter of the semicircle in the middle-frequency range) of the cell with the MCP/PVA hybrid separator is smaller. The lower interfacial resistance of the MCP/PVA hybrid separator can be understood by the good compatibility between the hydroxyl-rich skeleton of PVA and polar electrolyte molecules.

**Figure 4:**
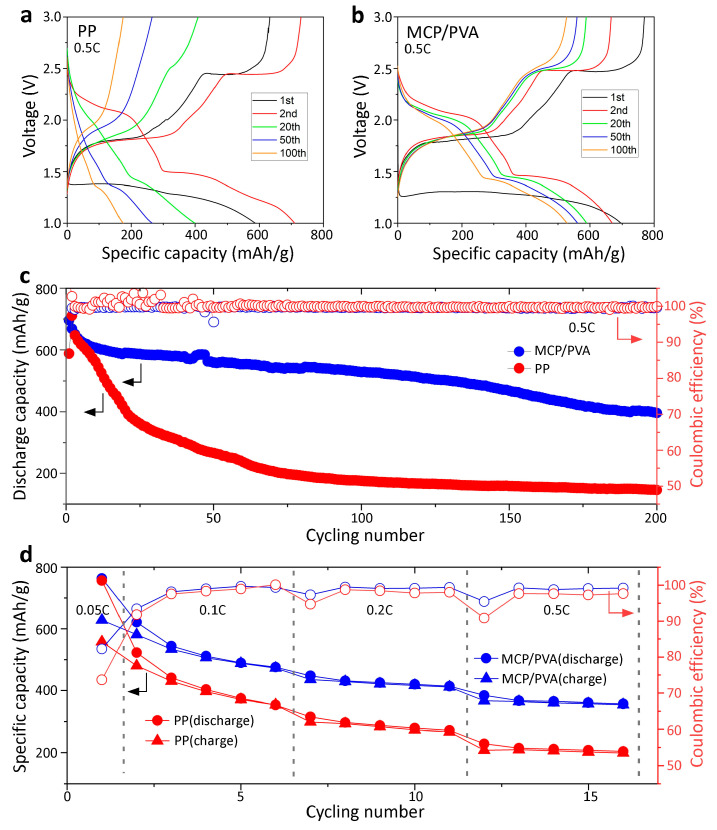
Cycling performances of the Li/FeS_2_ batteries with different separators. Charge and discharge curves of cells with (**a**) PP and (**b**) MCP/PVA separators at a current density of 0.5 C. (**c**) Long-term cycling performance at 0.5 C. (**d**) Cycling performance at small current densities.

To study the electrochemical performance of the hybrid separator in Li/FeS_2_ batteries, coin cells assembled with the commercial PP and MCP/PVA separators were evaluated. Figure 4a shows the cycling performance of the cell assembled with PP at a current density of 0.5 C. In the first discharge cycle, a voltage plateau at about 1.4 V can be identified, with a discharge-specific capacity of 587 mAh/g corresponding to the lithiation of FeS_2_ into Li_2_S and Fe [13,34]. In the following charge process, two voltage plateaus at around 1.7 V and 2.4 V, corresponding to $Fe+{Li}_{2}S\to FeS+2Li$ and $8{Li}_{2}S\to S_{8}+16Li$ reactions, can be observed [8]. As the cycling measurements proceed, the specific capacity of the cell decreases dramatically to ~200 mAh/g after 100 cycles. And both charge voltage plateaus vanish, especially the one at 2.4 V, which disappears after 50 cycles. According to previous mechanism studies, the significant loss in capacity is attributed to the severe shuttle effect of the polysulfides produced at 2.4 V, which dissolve in ether-based electrolytes and transfer through the PP separator to react with Li metal anodes [10]. On the other hand, for the cell assembled with the MCP/PVA hybrid separator, an initial discharge-specific capacity of ~700 mAh/g was achieved, as shown in Figure 4b. The much higher initial discharge capacity of the hybrid separator as compared with the PP separator can be ascribed to the uniform lithium-ion flux guided by the nanopores at a high discharge rate of 0.5 C. Excitingly, the cycling stability of the cell with the hybrid separator is also better, with a capacity retention of ~70% after 100 cycles at 0.5 C. Specifically, the charge voltage plateau at 2.4 V, corresponding to the conversion process of Li_2_S to S [8,17], can be observed throughout the process, indicating an effective trapping effect of polysulfides by the hybrid separator. Long-term cycling measurements were also performed, as shown in Figure 4c. For the cell with the commercial PP separator, the specific capacity drops rapidly to ~400 mAh/g after 20 cycles and ~200 mAh/g after 75 cycles at a current density of 0.5 C. However, the electrochemical performance of the Li/FeS_2_ cells was greatly enhanced with the application of the MCP/PVA separator, which can still deliver a discharge capacity of 500 mAh/g after 100 cycles and ~400 mAh/g after 200 cycles at a current density of 0.5 C. Moreover, in spite of the capacity decay, the coulombic efficiencies in the cycling test of both cells were close to ~100%, which can be explained by the inhibition of the shuttle effect with the increase in the current rate in the Li/S [35] and Li/FeS_2_ [13] systems. Therefore, to better investigate the shuttle effect mitigation performance of the different separators, the Li/FeS_2_ batteries were also tested at small current rates, as shown in Figure 4d. In the initial cycle at a current rate of 0.05 C, both cells displayed a discharge-specific capacity of ~760 mAh/g; however, a charge-specific capacity of only 628 mAh/g and 557 mAh/g with a coulombic efficiency of 82% and 70% was obtained for the hybrid separator and the PP separator, respectively. The higher coulombic efficiency of the cell with the hybrid separator suggested improved shuttle effect mitigation. Similarly, in the following 15 cycles at a current rate from 0.1 C to 0.5 C, the coulombic efficiencies of the cell with the hybrid separator (average CE of 98%) outperformed those of the one with the PP separator (average CE of 92%). Therefore, it can be concluded that the improvement of the cycling performance for the hybrid separator is due to the improved shuttle effect mitigation. Nevertheless, both cells experienced a serious capacity decay at small current rates due to the lack of surface modification on FeS_2_, and this should be further improved in future work.

**Figure 5:**
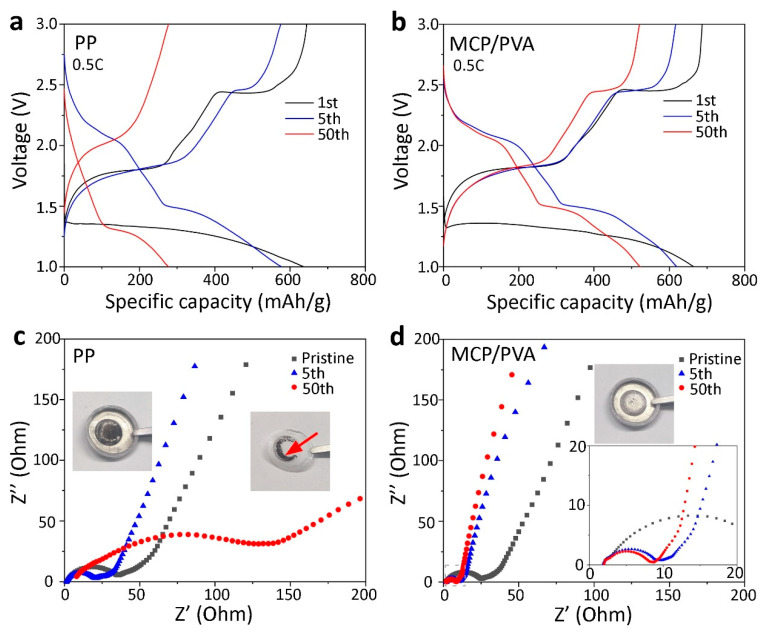
Charge and discharge curves of Li/FeS_2_ batteries with (**a**) PP and (**b**) MCP/PVA separators at 0.5 C in different cycles. EIS results of the cells with (**c**) PP and (**d**) MCP/PVA separators after different cycles. The red arrow in (**c**) refers to the black substances on the separator.

In order to understand the interfacial degradation of the cells with different separators, EIS measurements were conducted after different cycles. Figure 5a,b show the voltage profiles of the cells with the commercial PP separator and the MCP/PVA hybrid separator in the 1st, 5th and 50th cycle at a current density of 0.5 C. EIS measurements were carried out before cycling and after the charging procedure of the 5th and 50th cycle. It was found that more than 500 mAh/g of capacity was retained by the cell with the hybrid separator, while the specific capacity of the cell with the commercial PP separator was less than 300 mAh/g after 50 cycles. As shown in Figure 5c,d, the R_ct_ value of the cell with the hybrid separator is smaller than that with the PP separator, suggesting a better electrolyte/electrode interfacial property in the pristine state. After five cycles, the R_ct_ value of both cells decreased obviously, and this can be understood by the enlarged electrolyte/FeS_2_ contact area due to the breakage of large commercial FeS_2_ particles, which is related to an activation process [17,36,37]. As the cycling tests proceeded, the effect of the activation process weakened and the interfacial reaction dominated in the determination of the R_ct_ values [38]. The significantly increased R_ct_ value for the cell with the PP separator implied the accumulation of inactive species on the electrode surface. This was evidenced by the inset photos in Figure 5c, where apparent black substances on both the lithium metal and the side of the separator facing it were observed after disassembling the cell [17]. These residues acted as a barrier to electrochemical reactions, leading to a slow kinetics in this case. In contrast, the R_ct_ value of the cell with the hybrid separator after 50 cycles remained relatively stable compared with that after 5 cycles, which suggested the inhibition of side reactions at the interface despite the capacity decay. Additionally, the extracted lithium metal was much brighter, demonstrating the suppression of the shuttle effect with the use of the hybrid separator.

**Figure 6:**
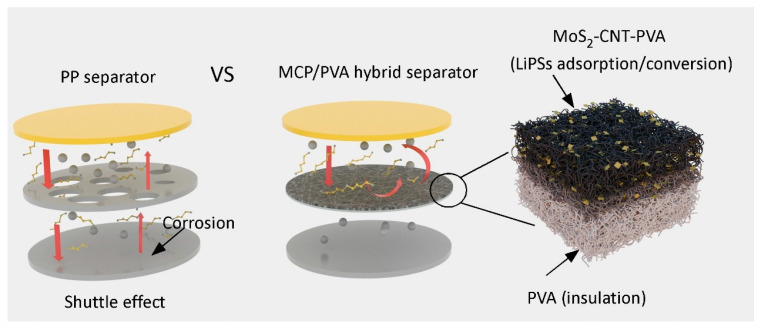
Schematic illustration of the working principle for the hybrid separator in Li/FeS_2_ batteries. The red arrows indicate the movement of LiPSs during cycling.

The schematic illustration in Figure 6 shows the working principle of the hybrid separator in Li/FeS_2_ batteries. In the cell with the conventional PP separator, the LiPSs produced in the cycling process can dissolve in electrolytes and diffuse through the large pores in the PP separator, resulting in capacity loss and surface corrosion. On the other hand, the cycling stability of Li/FeS_2_ batteries can be greatly enhanced with the application of the MCP/PVA hybrid separator. Specifically, the electrospun pure PVA layer with uniform nanopores in the bottom facilitates uniform lithium-ion flux and provides electrical insulation, while the top MCP layer attached to the FeS_2_ cathode is able to effectively adsorb LiPSs with the hydroxyl-rich PVA skeleton and the introduced MoS_2_ and CNTs act as the catalytic sites for LiPS conversion, realizing high utilization of LiPSs and improved cycling stability.

## 4. Conclusions

In this work, we successfully synthesized MCP/PVA hybrid films by the electrospinning technique and investigated their possible application as separators in rechargeable Li/FeS_2_ batteries. The hybrid separator exhibited a highly porous structure with excellent electrolyte wettability. Electrochemical measurements of Li/FeS_2_ batteries assembled with the hybrid separator delivered a high capacity of 400 mAh/g after 200 cycles at a current density of 0.5 C. The greatly improved cycling performance can be attributed to the unique layer structure of the hybrid film, with the bottom PVA layer providing electrical insulation and the top MCP layer adsorbing and catalyzing the conversion of LiPSs. In summary, this study provides a novel approach to inhibiting the shuttle effect in Li/FeS_2_ batteries by constructing a PVA-based electrospun hybrid separator, which can also be applied in sulfur-based rechargeable lithium metal batteries.

## Data Availability

Data are contained within the article and the Supplementary Materials.

## Supplementary Materials

The following supporting information can be downloaded at: https://www.mdpi.com/article/10.3390/polym16070921/s1, Figure S1: EDS results for large particles; Figure S2: Electrolyte contact-angle test on pure PVA membrane.
