# Supplementary material for: Electrospun MoS2-CNTs-PVA/PVA Hybrid Separator for High-Performance Li/FeS2 Batteries
Source: Polymers (Basel). 2024 Mar 27;16(7):921. doi: 10.3390/polym16070921 (PMC11013839; doi:10.3390/polym16070921)
Supplement: Supplementary file 1 [file polymers-16-00921-s001.zip › polymers-2930967-supplementary.pdf]

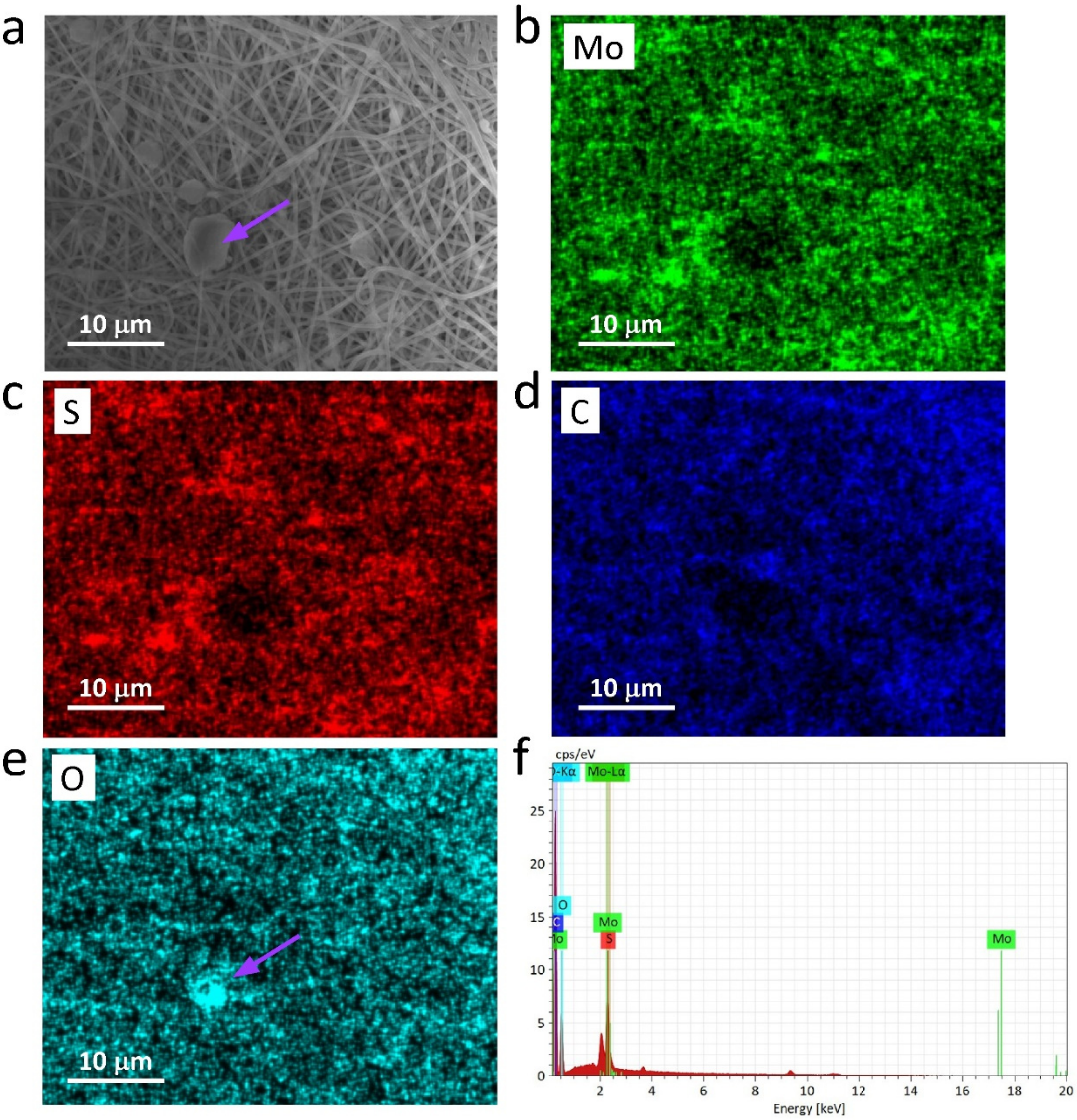

Figure S1. EDS results of large particles.

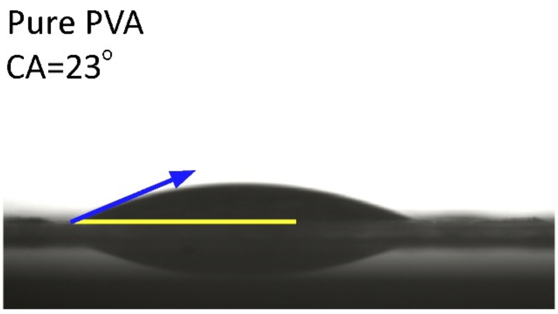

Figure S2. Electrolyte contact angle test on pure PVA membrane.
